# Promoting Healthy Environments In Afterschool Settings: The LiveWell Greenville Afterschool Initiative

**DOI:** 10.5888/pcd15.180164

**Published:** 2018-11-21

**Authors:** Karen A. Kemper, Sarah P. Pate, Alicia R. Powers, Melissa Fair

**Affiliations:** 1 Department of Public Health Sciences, Clemson University, Clemson, South Carolina; 2 LiveWell Greenville, Greenville, South Carolina; 3 Kidnetics, Greenville, South Carolina; 4 Supplemental Nutrition Assistance Program–Education, Alabama Cooperative Extension, Auburn University, Alabama; 5 Institute for the Advancement of Community Health, Furman University, Greenville, South Carolina

## Abstract

**Introduction:**

LiveWell Greenville, a multi-organization community coalition, launched an initiative in 2011 to help afterschool programs promote environments that decrease the risk of obesity among children and adolescents. The objective of this study was to describe changes in nutrition and physical activity environments, policies, and practices among 37 afterschool programs after their participation in the LiveWell Greenville Afterschool Initiative.

**Methods:**

The study used a nonexperimental, pre- and postsurvey design. The survey was based on the Nutrition and Physical Activity Self-Assessment for Child Care questionnaire and modified for afterschool settings. Items addressed sedentary time, physical activity time, availability of sugar-sweetened beverages, sweet and salty snack consumption, fruit consumption, staff behaviors, and policies that support nutrition and physical activity practices. The self-assessment survey was completed by an afterschool program supervisor at each site. The 9-month intervention consisted of program staff members’ completing the pre-assessment and goal-setting worksheet, receiving technical support and training from LiveWell Greenville staff, attending networking meetings about nutrition and physical activity promotion strategies, and completing a postassessment.

**Results:**

We found significant positive changes in afterschool environments in the type and frequency of snacks offered, duration of children’s sedentary time, staff behaviors that supported healthy nutrition and physical activity practices, and education provided to staff, children, and parents.

**Conclusion:**

We found the LiveWell Greenville Afterschool Initiative, which involved self-assessment, goal setting and technical support, to be a successful strategy to change nutrition and physical activity environments in afterschool settings.

## Introduction

Childhood obesity is a health problem in the United States and is associated with numerous chronic illnesses as well as social stigma and a diminished quality of life (1–5). Obesity prevalence has doubled among children and more than quadrupled among adolescents since the 1980s (6, 7). Although prevalence rates among children and adolescents did not increase significantly from 2011 to 2014, rates are high, at 17% (8). Environmental, policy, and systems changes that support healthy weight among children are a priority (9). Of the approximately 10 million students served by afterschool programs each day, 61% are in elementary school, 23% in middle school, and 16% in high school (10). Children accumulate only 13 to 24 minutes of moderate-to-vigorous activity per day in afterschool settings, and the snacks provided frequently consist of processed foods high in sugar and fat (11–13). To provide guidance to afterschool programs about best practices for healthier environments for children, the national Healthy Out-of-School Time Coalition (HOST) established the healthy eating and physical activity (HEPA) standards in 2011 (14).

Before the release of the HEPA standards, a community coalition, LiveWell Greenville (LWG), was formed in a southeastern county in the United States to address growing concerns about childhood obesity. LWG focuses on creating environmental, policy, and systems changes in partnering organizations to prevent childhood obesity, and it selected afterschool settings as a priority. LWG conducted formative research with afterschool providers to determine interests and needs in making changes that support the HEPA standards. On the basis of representatives’ input, LWG formed the Out-of-School Time (OST) workgroup in 2011 to develop and launch the LWG Afterschool Initiative to assist afterschool programs in adopting nutrition and physical activity practices that promote healthier environments for their children. As of 2018, the LWG Afterschool Initiative is an ongoing program. The objective of this study was to examine changes in nutrition and physical activity environments, policies, and practices in afterschool programs that participated in the LWG Afterschool Initiative from 2011 through 2014.

## Methods

This study used a nonexperimental, pre- and postsurvey design to examine changes in nutrition and physical activity environments, policies, and practices among afterschool programs participating in the LWG Afterschool Initiative. We collected self-reported data from 2011 through 2014, using an online self-assessment survey and a goal-setting worksheet completed by an afterschool supervisor at each site, and we analyzed data in 2014. The study received institutional review board approval from Furman University in 2011.

The LWG OST workgroup developed a 9-month intervention modeled after the Nutrition and Physical Activity Self-Assessment for Child Care (NAP SACC) program. This model was chosen because of its emphasis on environmental and policy changes that have the potential to influence nutrition and physical activity behaviors. The NAP SACC program was developed by the North Carolina Division of Public Health in 2002 to assess best practices and promote healthy nutrition and physical activity environments in child care settings (15,16). The NAP SACC program uses a self-assessment survey intended to be completed by child care center directors to assess their facility policies and practices based on key areas of nutrition and physical activity (16). The self-assessment allows directors to identify strengths and limitations of the facility and to establish goals that they feel ready and able to address (17). The original NAP SACC program includes 4 steps: 1) self-assessment, 2) goal setting, 3) training, and 4) reassessment (16). The NAP SACC program has improved nutrition and physical activity outcomes in child care settings (16–18).

The self-assessment step is a key component of the intervention process (17). The NAP SACC self-assessment questionnaire is designed to introduce nutrition and physical activity best practices and to allow the representatives of a child care setting to assess their program relative to those standards (17). The questionnaire includes 9 nutrition areas and 6 physical activity areas, each with 4 numerical response-option categories, ranging from 1 (the minimal standard) to 4 (best practice), based on national standards (16). In an examination of the NAP SACC instrument’s reliability and validity (19), investigators determined that the NAP SACC instrument is a reasonably accurate and stable tool for use in child care interventions, but they recommended less subjective measures, such as environmental audits conducted by outside observers, for assessing the impact of an intervention.

The LWG-OST workgroup modified the original NAP SACC self-assessment instrument to better reflect best practices for afterschool settings. The original instrument was designed for day care facilities, which typically serve preschool-aged children for an extended period of the day. Afterschool programs typically serve school-aged children for a briefer period after dismissal of a regular school day. We modified the NAP SACC instrument by removing nutrition items that were less relevant to most of our afterschool settings than to day care facilities (eg, serving of multiple meals, serving meats, beans, and vegetables) and by adapting response options to reflect afterschool program daily duration and weekly frequency (eg, frequency per day for foods, duration per day of physical activity, frequency per week of menu items). The original NAP SACC instrument consists of 37 nutrition items and 17 physical activity items. Our self-assessment survey consists of 27 nutrition items and 17 physical activity items. We use a Likert-type scale response option format, ranging from 1 (minimal performance) to 5 (best practice performance). Because our program was launched before the release of the HEPA afterschool standards, our nutrition and physical activity best practices were derived from NAP SACC best practices for day care settings. The survey includes items about physical activity time and practices, sedentary time, physical activity and nutrition environments, sugar-sweetened beverages, sweet and salty snacks, fruit snacks, whole-grain snacks, and staff member’s behaviors and policies that support nutrition and physical activity practices. The survey also includes questions on demographic characteristics. Validation of our modified NAP SACC instrument was not possible with the time and resources that were available to us.

We categorized afterschool programs based on program setting and administrative structure. We classified programs as school affiliated if they were located at school campuses and overseen by school administration; non–school affiliated if they were not located on school campuses and not overseen by school administrations; and hybrid if they were located on school campuses but were not overseen by school administration. We reviewed afterschool program goals and strategies developed as part of the intervention, using a paper-and-pencil goal-setting worksheet completed by each afterschool representative and collected by LWG staff members.

### Participants and sampling

The study was conducted in Greenville County, South Carolina. The county has an estimated population of 498,766; 24.2% are younger than 18 years; 76.7% are white; 18.6% are African American; the median household income in 2012–2016 was $52,595; and 15.2% of the population lives below the federal poverty level (20). A coalition partner, the United Way of Greenville’s Building Opportunities in Out of School Time (BOOST), provided a list of 194 afterschool programs registered with their OST network. Participants were recruited through coalition members (eg, United Way, faith-based afterschool programs, public school extended-care programs, summer camps, city and county recreation departments). To be eligible to participate in the intervention, programs had to provide an afterschool program to school-aged (kindergarten through grade 12) children in Greenville County.

A convenience sample of 37 afterschool programs participated in the intervention and consented to be part of the study. Afterschool programs were enrolled during a 3-year period. During Year 1 of the afterschool intervention, 8 programs were enrolled in the intervention, during Year 2, 16 programs were enrolled, and during Year 3, 13 programs were enrolled. Participating programs were required to complete a memorandum of agreement with LWG that clarified what the program consisted of and what each party agreed to do as part of the initiative (ie, complete pre- and postassessment, set goals, and attend training sessions). Program leaders also were asked to review and, if in agreement, sign informed consent documents approved by an institutional review board of Furman University. Program recruitment was launched before the start of the new school year, and the intervention began during the first 1 to 3 months of the school year. The initial self-assessment instrument was completed by each program’s supervisor shortly after enrollment and again approximately 9 to 12 months after enrollment. Supervisors were encouraged to consult with program staff to answer the survey questions.

### Intervention

The LWG OST workgroup adapted the NAP SACC program model for use in an afterschool environment. The LWG intervention process included 5 steps: 1) self-assessment (a program orientation meeting, completion of a pre-self-assessment survey, 2) planning (goal-setting meeting with LWG staff), 3) implementation (training in a nutrition and physical activity curriculum and optional monthly networking sessions), 4) evaluation (a post–self-assessment and follow-up meetings with LWG staff), and 5) celebration (annual meeting of all participating afterschool programs).

Resources provided to each afterschool program consisted of a Comprehensive Approach To Child Health (CATCH) Kids Club Afterschool curriculum and associated play equipment, an online tool kit developed by the LWG team, and technical assistance provided by LWG staff. The CATCH curriculum combines nutrition education and physical activity for an elementary- and middle-school–aged population (21). An online tool kit was also available to all afterschool program participants. The tool kit contained information that outlined the steps and materials used in the LWG afterschool initiative, links to local and national resources covered in networking sessions, and detailed information about nutrition and physical activity best practices for afterschool settings.

During the orientation meeting, participants were introduced to the components of the LWG afterschool intervention, technical support staff, self-assessment survey, training workshop, and online tool kit resources. Programs were encouraged to include supervisors and staff members in orientation. After completion of the self-assessment survey, an LWG staff member met with the designated afterschool program representatives (typically the program supervisor and a staff member) to review the self-assessment data and to identify areas that the afterschool program wanted to address. The afterschool representatives, with assistance from the LWG staff member, completed a goal-setting worksheet. Each afterschool program was asked to identify at least 1 nutrition goal, 1 physical activity goal, and 1 policy goal that they would like to change during the next 9- to 12-month period.

Training opportunities were available to all participating afterschool program staff. Monthly networking sessions also were offered to the participating afterschool programs. Networking sessions consisted of brief informational components, activity or nutrition demonstrations, and opportunities for attendees to discuss what they were doing at their programs and areas in which they needed support. At least 1 representative of each afterschool program was required to participate in the CATCH curriculum training.

### Data analysis

Pre- and posttest responses of the modified NAP SACC survey were exported from an online data collection system (SurveyMonkey) into SPSS version 22 (IBM Corporation). Selected items were reverse scored so that higher scores reflect preferred practices and policies. Data from afterschool program goal worksheets were reviewed, coded for goal type, and added to the SPSS data file. All statistical analysis was performed in SPSS version 22. We used paired *t* tests to determine significant differences (*P* < .05) between pre- and posttest responses on the modified NAP SACC survey. We used the general linear model to examine interaction effects of program type on responses to the modified NAP SACC survey. We used the Bonferroni method to adjust α values for post hoc comparisons. After the pre- and posttest analysis of nutrition and physical activity items, we made a nonstatistical comparison of the 6 most frequently listed planning goals and the survey questions corresponding to practices related to those goals.

## Results

Of the 37 programs enrolled in the LWG afterschool intervention, 30 (81%) programs completed both pre-and postassessments. The afterschool programs that participated in the intervention consisted of school-affiliated (n = 11) programs, county or city recreation programs (n = 9), faith-based programs (n = 2), for-profit programs (n = 2), and nonprofit programs (n = 13). Programs varied in size; most (n = 30) served 25 to more than 100 children each day (Table 1). The 7 programs that did not complete a postassessment had enrolled in the second (n = 3) and third (n = 4) year of the intervention and were school-affiliated programs (n = 3), non–school-affiliated programs (n = 3), and hybrid programs (n = 1).

**Table 1:** Characteristics of Afterschool Programs (n = 37) Participating in the LiveWell Greenville Afterschool Initiative, Greenville, South Carolina, 2011–2014^a^

Characteristic	No. (%)
**No. of children served by program^b^**	
<25	4 (10.8)
25–50	12 (32.4)
51–100	12 (32.4)
>100	6 (16.2)
Missing data	3 (8.1)
**Median income of families served by program, $^b^**	
<25,000	12 (32.4)
25,000–34,999	12 (32.4)
35,000–49,999	3 (8.1)
50,000–74,999	3 (8.1)
>75,000	1 (2.7)
Missing data	6 (16.2)
**Site type^c^**	
School affiliated	11 (29.7)
Hybrid	8 (21.3)
Non–school affiliated	18 (48.6)
**Grade level served by program^b^**	
Elementary school grades only	21 (56.8)
Middle school grades only	1 (2.7)
Elementary and middle school grades	15 (40.5)
**Cohort year^d^**	
Year 1	8 (21.6)
Year 2	16 (43.2)
Year 3	13 (35.1)

a Intervention was designed to measure changes in nutrition and physical activity environments, policies, and practices.

b Self-reported by an afterschool program supervisor.

c Site type based on the location of the program and the governing organization. School-affiliated programs were implemented at a school and directed by school staff (n = 11 elementary schools). Non–school-affiliated programs were implemented at locations other than a school and directed by nonschool staff (n = 9 county recreation or community centers, 4 private programs, and 5 YMCA programs). Hybrid programs were implemented at a school but directed by nonschool staff (5 Community in Schools programs, 2 YMCA programs, 1 nonprofit partnership).

d Cohort year was assigned according to year of enrollment in the LiveWell Greenville Afterschool Initiative. Enrollment occurred in fall 2011 (Year 1), fall 2012 (Year 2), and fall 2013 (Year 3).

Study participants reported high scores (4 or 5) for several practices at baseline. Most sites reported offering sugar-sweetened beverages fewer than 1 time per week or never (n = 33, 92%), offering active play for 30 or more minutes per day (n = 27, 73%), and offering outdoor play 3 or more days per week (n = 32, 91%). Programs were less likely to report practices such as offering fruit 3 or more times per week (n = 16, 46%), offering high-fiber snacks 3 or more times per week (n = 12, 32%), offering 100%-fruit juice fewer than 1 time per week or never (n = 14, 40%), and limiting sedentary time (children seated >30 minutes at a time) to less than 1 time per week or never (n = 10, 28%).

In the self-assessment survey for the 30 programs completing the pre- and postintervention assessment, we found significant positive differences between pre- and posttest mean scores for nutrition items on decreasing consumption of sweet or salty snacks (*P* = .02), increasing fruit offered (*P* < .001), encouraging children to try new foods or less-flavored foods (*P* = .02), having drinking water available outdoors (*P* = .005), providing nutrition education to children (*P* = .01) and parents (*P* = .009), providing nutrition guidelines to parents (*P* = .01), and developing and following written nutrition policies (*P* < .001) (Table 2). For physical activity items, we found significant positive differences between pre- and posttest mean scores for decreasing sedentary time (*P* < .001), increasing availability of fixed play equipment (*P* = .02) and indoor portable play equipment (*P* = .001), increasing staff participation in physical activity with the children (*P* = .003), providing physical activity education to the children (*P* = .01) and parents (*P* = .02), and developing and following written policy for physical activity (*P* < .001). We found no significant differences between pre- and posttest responses across program types.

**Table 2:** Self-Reported Preintervention and Postintervention Scores^a^ on the Modified NAP SACC Questionnaire^b^, Participants in LiveWell Greenville Afterschool Initiative, Greenville, South Carolina, 2011–2014^c^

Item	No. of Programs That Completed Both Assessments	Preintervention Score,^a^ Mean (SD)	Postintervention Score,^a^ Mean (SD)	*t* Test (*df*) [*P* Value] For Difference Between Scores^d^
**Nutrition**				
Sweet or salty snacks offered	30	3.5 (0.9)	4.0 (0.9)	−2.5 (29) [.02]
Sugar-sweetened beverages offered	30	4.6 (0.9)	4.5 (0.9)	0.33 (29) [.74]
High-fiber, whole-grain foods offered	30	3.1 (1.1)	3.5 (0.9)	−1.9 (29) [.06]
Fruit (not juice) offered	28	3.2 (1.4)	3.9 (0.9)	−3.0 (27) [.006]
Fruit that is canned in own juice, fresh, frozen offered	28	2.1 (1.2)	2.2 (1.2)	−0.2 (27) [.83]
100% Fruit juice offered	29	2.9 (1.5)	3.3 (1.5)	−1.8 (28) [.09]
Snack menu variety used	29	3.2 (1.1)	3.2 (1.0)	−0.2 (28) [.88]
Food used to encourage positive behavior	29	4.2 (0.9)	4.4 (0.9)	−0.8 (28) [.42]
Removing food if child feels full	29	2.7 (1.5)	3.0 (1.5)	−1.4 (28) [.18]
Serving seconds if child still hungry	29	2.9 (1.7)	2.9 (1.7)	0.1 (28) [.93]
Encouraged children to try new or less-favored foods	29	4.0 (1.1)	4.4 (0.7)	−2.6 (28) [.02]
Staff join children at table	29	3.1 (1.6)	3.7 (1.2)	−2.0 (28) [.05]
Staff consume same food and drinks	28	3.2 (1.5)	3.5 (1.4)	−1.0 (27) [.31]
Staff eat/drink less healthy	28	4.1 (0.8)	4.3 (0.8)	−0.8 (27) [.41]
Staff talk informally with children about healthy foods	29	3.6 (0.9)	3.9 (0.9)	−1.3 (28) [.21]
Holidays celebrated with healthy food	29	3.0 (1.1)	3.2 (1.1)	−1.1 (28) [.28]
Fundraising uses only nonfood items	18	0.8 (1.3)	0.8 (1.5)	0 (17) [>.99]
Milk quality served	27	2.4 (1.3)	2.4 (1.3)	−0.1 (26) [.90]
Drinking water available outdoors	27	2.0 (1.2)	2.6 (1.3)	−3.1 (26) [.005]
Drinking water available indoors	29	3.4 (0.7)	3.6 (0.6)	−1.2 (28) [.23]
Vending machines present in facility	28	1.7 (0.7)	1.6 (0.5)	0.6 (27) [.57]
Training for staff provided	29	3.6 (1.5)	4.0 (1.3)	−1.1 (28) [.27]
Nutrition education for children provided	29	2.4 (1.4)	3.2 (1.4)	−2.6 (28) [.01]
Support for healthy nutrition displayed	28	2.0 (0.9)	2.8 (0.7)	−3.6 (27) [.001]
Nutrition education for parents offered	28	2.3 (1.4)	3.4 (1.7)	−2.8 (27) [.009]
Guidelines for holiday foods offered to parents	27	1.6 (1.1)	2.2 (1.4)	−2.7 (26) [.01]
Written nutrition policy developed and followed	27	1.9 (1.3)	3.1 (1.2)	−4.5 (26) [<.001]
**Physical activity**				
Time for active play provided	30	3.8 (0.8)	4.1 (0.6)	−2.3 (29) [.03]
Teacher-led physical activity provided	30	3.7 (1.2)	4.0 (0.7)	−1.5 (29) [.13]
Outdoor active play provided	28	4.5 (0.6)	4.6 (0.6)	−1.8 (27) [.08]
Children not seated for more than 30 minutes at a time	28	2.6 (1.4)	4.1 (1.3)	−4.2 (27) [<.001]
Television and video time provided	29	3.9 (0.7)	4.0 (0.9)	−1.0 (28) [.33]
Outdoor play space quality	29	3.4 (0.7)	3.5 (0.7)	−0.4 (28) [.68]
Indoor play space quality	30	3.4 (0.9)	3.7 (0.6)	−1.7 (29) [.11]
Fixed play equipment available	28	2.9 (1.1)	3.3 (1.1)	−2.5 (27) [.02]
Outdoor portable play equipment available	26	2.8 (1.1)	3.1 (1.1)	−1.0 (25) [.33]
Indoor portable play equipment available	29	3.0 (0.8)	3.4 (0.7)	−3.5 (28) [.001]
Active play time withheld for misbehavior	29	2.1 (0.7)	2.4 (0.7)	−1.9 (28) [.06]
Staff encourage and participate in active play	29	3.3 (0.8)	3.8 (0.5)	−3.2 (28) [.003]
Training for staff provided	29	3.6 (1.3)	4.1 (1.0)	−1.9 (28) [.07]
Physical activity education for children provided	28	2.5 (1.6)	3.4 (1.3)	−2.7 (27) [.01]
Support for physical activity displayed	30	2.0 (1.2)	2.1 (0.8)	−0.4 (29) [.70]
Physical activity education offered to parents	26	2.0 (1.5)	3.1 (1.7)	−2.6 (25) [.02]
Written policy developed and followed	27	1.8 (1.1)	2.9 (1.2)	−4.6 (26) [<.001]

Abbreviations: *df*, degrees of freedom; NAP SACC, Nutrition and Physical Activity Self-Assessment for Child Care; SD, standard deviation.

a Mean scores from Likert-type response option categories ranged from 1 (minimal performance) to 5 (best practice performance). Increases in mean scores indicate improvements in nutrition and physical activity practices and policies.

b NAP SACC self-assessment questionnaire was modified to accommodate afterschool setting factors such as length of day, frequency per week, types of meals served (ie, snacks vs meals).

c Intervention was designed to measure changes in nutrition and physical activity environments, policies, and practices.

d Paired *t* tests were performed to determine differences between pre- and postintervention mean scores; significance set at <.05.

Goal worksheets for 25 programs were available for review at the time of the posttest assessment; from them, we identified 21 goal categories. The worksheets indicated 2 to 10 goals for each program. Of the 21 goal categories, 4 were selected by 15 or more of the programs: increasing fruit and vegetable consumption (n = 18 programs), increasing nutrition education to children (n = 17 programs), decreasing sedentary time (n = 15 programs), and developing written policies for nutrition and physical activity (n = 23 programs). In the comparison of the 6 most frequently listed planning goals, we found significant changes in mean scores for survey questions on practices related to 5 goals (Table 3).

**Table 3:** Goals Most Frequently Selected by Afterschool Programs (n = 25^a^) and Mean Score^b^ for Corresponding Item in NAP SACC,^c^ Participants in LiveWell Greenville Afterschool Initiative, Greenville, South Carolina, 2011–2014^d^

Goal Selected by Program	No. (%) of Sites That Selected Goal	Corresponding Modified NAP SACC Survey Item^c^	Preintervention Score–Postintervention Score^b^ (*P* Value^e^)
Increase fruit and vegetable offered	18 (72)	Fruit (not juice) provided	3.2–3.9 (.006)
Increase nutrition education for children	17 (68)	Nutrition education for children provided	2.4–3.2 (.01)
Increase physical activity time	7 (28)	Time for active play provided	3.8–4.1 (.03)
Decrease sedentary time	15 (60)	Children not seated for more than 30 minutes at a time	2.6–4.1 (<.001)
Implement Coordinated Approach to Child Health games	10 (40)	Teacher-led physical activity provided	3.7–4.0 (.13)
Develop a written policy for physical activity and nutrition	23 (92)	Written nutrition policy followed	1.9–3.1 (<.001)
		Written physical activity policy followed	1.8–2.9 (<.001)

Abbreviation: NAP SACC, Nutrition and Physical Activity Self-Assessment for Child Care.

a Goal worksheets for 25 of the 37 programs were available at the time of the posttest assessment.

b Mean scores were derived from a Likert-type response scale ranging from 1 (minimal performance) to 5 (best practice performance). Increases in mean scores indicate improvements in nutrition and physical activity practices and policies.

c NAP SACC questionnaire was modified to accommodate afterschool setting factors such as length of day, frequency per week, types of meals served (ie, snacks vs meals).

d Intervention was designed to measure changes in nutrition and physical activity environments, policies, and practices.

e Paired *t* tests were performed to determine differences between pre- and postintervention mean scores (*P* < .05).

## Discussion

Our study results indicated that several aspects of the nutrition and physical activity environments (active time, sedentary time, and snack and beverage offerings) improved after participation in the LWG Afterschool Initiative. We found that afterschool program environments could be positively shaped to increase healthy behaviors and practices among children and staff. The self-assessment survey and goal-setting meetings were used by afterschool providers to select nutrition and physical activity practices or policies that they wanted to change.

Other studies (12,22,23) also found that the nutritional quality of snacks improved during participation in interventions that emphasized best practices. These studies observed increases in the weekly frequency of servings of fruits and decreases in weekly servings of sugar-sweetened beverages and salty snacks (12,22,23). Another study (24) observed that afterschool use of HEPA standards for program planning was positively associated with improvements in the amount and type of physical activity offered.

The LWG Afterschool Initiative implemented several public health services recommended by the Centers for Disease Control and Prevention (25) to promote environmental changes in afterschool settings: creating community partnerships to identify and solve health problems in the community; using self-assessment to “inform, educate, and empower” afterschool program providers to address childhood obesity prevention; and encouraging afterschool providers to develop policy to support healthy environments for their children.

Since the launching of the LWG Afterschool Initiative in 2011, several internet-based resources emerged to support afterschool programs interested in adopting HEPA standards (eg, Harvard University’s Out of School Nutrition and Physical Activity Initiative [OSNAP] [26]; Alliance for a Healthier Generation Out-of-School Time program [27]; Policy to Practice in Youth Programs [P2YP] [28]). These programs mirror the best-practice–based model for self-assessment, goal setting, and resource assistance that was effective in our LWG Afterschool Initiative and provide practitioners and policy makers with low-cost access to tools to promote healthy environments in afterschool programs.

More research is needed to optimize the positive health impact of afterschool programs on children’s well-being. Identifying the most effective, sustainable, and cost-effective strategies and collaborations are important in determining the extent to which afterschool programs can successfully adopt and institutionalize the best-practice HEPA standards in afterschool settings (22,29).

Our study was limited by the absence of a control or comparison group that would allow for a more thorough examination of the intervention’s influence. We could not account for the influence of larger statewide or national childhood obesity prevention initiatives that could have influenced afterschool program providers. Our use of self-report measures also introduced a weakness in validity, and social desirability could have influenced self-report measures. Although Benjamin et al (19) found the original NAP SACC instrument to be reliable and valid overall, they also noted that self-report scores for best practices were consistently higher than those observed with objective measures collected by external sources. In addition, we used a coalition-based, self-selected convenience sample of moderate size, and this could limit the generalizability of the findings to other afterschool programs. Future studies that examine the sustainability of the changes observed in this study would be useful in assessing the long-term success of this type of initiative.

The LWG Afterschool Initiative resulted in positive changes in mean scores for several nutrition and physical activity practices among afterschool program participants. These changes resulted in healthier environments for children who attended these afterschool programs. The LWG Afterschool Initiative also demonstrated that the intervention model of self-assessment, goal setting, technical assistance, evaluation, and celebration was successful in afterschool settings.
